# The chemotype core collection of genus *Nicotiana*

**DOI:** 10.1111/tpj.15745

**Published:** 2022-04-07

**Authors:** Margit Drapal, Eugenia M. A. Enfissi, Paul D. Fraser

**Affiliations:** ^1^ Department of Biological Sciences Royal Holloway University of London Egham UK

**Keywords:** *Nicotiana*, metabolite analysis, pan‐metabolome, chemotype core collection, tobacco, chlorogenic acid, terpenoids

## Abstract

Sustainable production of chemicals and improving these biosources by engineering metabolic pathways to create efficient plant‐based biofactories relies on the knowledge of available chemical/biosynthetic diversity present in the plant. *Nicotiana* species are well known for their amenability towards transformation and other new plant breeding techniques. The genus *Nicotiana* is primarily known through *Nicotiana tabacum* L., the source of tobacco leaves and all respective tobacco products. Due to the prevalence of the latter, *N. tabacum* and related *Nicotiana* species are one of the most extensively studied plants. The majority of studies focused solely on *N. tabacum* or other individual species for chemotyping. The present study analysed a diversity panel including 17 *Nicotiana* species and six accessions of *Nicotiana benthamiana* and created a data set that effectively represents the chemotype core collection of the genus *Nicotiana*. The utilisation of several analytical platforms and previously published libraries/databases enabled the identification and measurement of over 360 metabolites of a wide range of chemical classes as well as thousands of unknowns with dedicated spectral and chromatographic properties.

## Significance Statement

A chemotype core collection for the genus *Nicotiana* was compiled. The metabolite composition of 17 *Nicotiana* species analysed was reproducible under different growth seasons and showed a rich source of chemical diversity. The compiled database catalogued over 360 identified metabolites and thousands of unknown chemical features. These data highlight *Nicotiana* species with enhanced levels of high‐value chemicals and of precursors utilised for pathway engineering of high‐value chemicals in a renewable manner.

## INTRODUCTION

The genus *Nicotiana* is most commonly known through one species, *Nicotiana tabacum* L., which is grown for tobacco leaves used for smoking and other tobacco products. *Nicotiana tabacum* is one of the most popular non‐food crops and grown in over 120 countries (Barla & Kumar, 2019). The top 10 producers worldwide produced >5.3 million metric tons of tobacco leaves in 2019 (Food and Agriculture Organization of the United Nations, 2021). Using tobacco for pleasurable effects has been a long tradition in pre‐Columbian America and other *Nicotiana* species were implemented for smoking purposes, e.g. *Nicotiana rustica* L., *Nicotiana repanda* Willdenow ex Lehmann, *Nicotiana attenuata* Torrey ex S. Watson and *Nicotiana quadrivalvis* Pursh (Charlton, 2004). The genus *Nicotiana* comprises 76 naturally occurring species in 13 sections, which are predominantly native to North and South America and Australia (Goodspeed, 1954; Knapp et al., 2004). A wide range of floral and vegetative morphology is present in *Nicotiana* species and accounts for their popularity as ornamental plants, e.g. *Nicotiana sylvestris* Spegazzini and Comes, *Nicotiana alata* Link and Otto and *Nicotiana langsdorffii* Weinmann (Knapp et al., 2004; Lewis, 2011). The diverse morphological range of *Nicotiana* species is reflected in the chemical diversity of the plants, which includes over 4000 comprehensively annotated metabolites in tobacco tissue (Rodgman & Perfetti, 2013). The compound classes include low‐molecular‐weight metabolites and macromolecules such as carbohydrates, proteins and amino acids, bases, hydrocarbons and lipids, phenolics, lignin, terpenoids and alkaloids. Many of these compounds deliver health‐promoting or medicinal benefits. Hence, *Nicotiana* plants have a long history of use as anaesthetics, narcotics and emollients, as medicinal components for treating ailments such as diarrhoea, burns and ulcerated abscesses and as toothpaste for whitening teeth (Charlton, 2004; Lewis, 2011). More recently, in the 1970s and 1980s, plant tissue culture experiments highlighted another use of *Nicotiana* plants as they can be more easily transformed and regenerated compared to other plant species (Horsch et al., 1985; Vasil et al., 1979). Particularly, *Nicotiana benthamiana* Domin and *N. tabacum* are amenable to nuclear and plastid transformation and have been used extensively for metabolic engineering (Molina‐Hidalgo et al., 2020). The many scientific and medicinal traits of *Nicotiana* promoted the extensive study of its genome, proteome, metabolome and phenotypic traits. Comparisons of ancestral and modern, as well as diploid and allopolyploid, species enable the elucidation of the evolution of polygenetic traits (Anssour et al., 2009; Sierro et al., 2014; Wu et al., 2006; Xu et al., 2017). Modern analytical techniques, such as liquid and gas chromatography coupled to mass spectrometry, facilitate the characterisation of biological active metabolites and the screening of a large number of samples for high‐value chemicals (Jassbi et al., 2017; Pinu et al., 2019). However, the wide quantitative range and chemical diversity of metabolites pose a significant challenge for the analysis of the pan‐metabolome, comprising core and dispensable metabolites of a species or genus (Halket et al., 2005; Medini et al., 2005). A more practical approach for the identification of the baseline metabolome and individual species with desired metabolic pathways is the compilation of a chemotype core collection (CCC) (Price et al., 2020). The combination of genetic and metabolic screening of a CCC with new plant breeding techniques can design *Nicotiana* species with improved pathways and metabolite levels as renewable biofactories in a sustainable bioeconomy (Molina‐Hidalgo et al., 2020). Hence, the present study generated a metabolite data set for the genus *Nicotiana*. To achieve this goal, a diversity panel was compiled with 17 representative *Nicotiana* species and six accessions of *N. benthamiana* from 12 *Nicotiana* sections and leaf material analysed with targeted and metabolite profiling techniques. The data generated show the range of over 360 identified metabolites representing the CCC of *Nicotiana* to date.

## RESULTS AND DISCUSSION

### Establishing the chemotype core collection of genus *Nicotiana*

For the assessment of the CCC of the genus *Nicotiana*, a diversity panel was analysed with different metabolomics tools. The *Nicotiana* species chosen for the diversity panel represent all sections of the *Nicotiana* genus, except for *Trigonophyllae* Goodspeed (Table 1), and were cultivated under glasshouse conditions to minimise environmental stress. The diversity panel was subjected to analysis with four different techniques on three platforms (LC‐high‐resolution MS [HRMS], GC‐MS and UPLC‐photodiode array detection [PDA]) to cover the extensive diversity of metabolites in the leaf material. Previous studies highlighted changes in the metabolic plasticity of leaves throughout the plant life‐cycle with significant changes at the reproductive stage (Li et al., 2016; Zhang et al., 2018). Hence, the two youngest fully expanded leaves were collected at the emergence of flower buds, to characterise the metabolome of each biological replicate. In addition, the diversity panel was cultivated twice under glasshouse conditions for a more robust representation of the metabolome. The metabolite data highlight that the chemical composition of the *Nicotiana* species panel was consistent over both growth seasons and emphasises their usefulness as biorefining feedstocks or a renewable plant‐based source of high‐value compounds (Figure S1). The three species of section *Tomentosae* Goodspeed – *Nicotiana tomentosiformis* Goodspeed (N479), *Nicotiana otophora* Grisebach (N406) and *Nicotiana tomentosa* Ruiz and Pavon var. *leguiana* (J.F. Macbr.) Goodspeed (N407) – were sampled before emergence of flowers to avoid a significant difference in sampling time. These three species have flowering times of 9 months to 2 years contrary to the average flowering time of the diversity panel, which was 2 months.

**Table 1:** *Nicotiana* diversity panel described with sample code used in the present study, species and section classification and phenotypic traits (germination time, flower emergence, leaf size and plant height)

Sample code	^a^	Species	*N. benthamiana* accession	*Nicotiana* Section	Germination (days after sowing)	Flower emergence		
						(days after germination)	Fully expanded leaf (cm)	Plant height (cm)
NIC528	c	*N. langsdorffii* Weinmann		*Alatae* Goodspeed	8	57	23	45
NIC44	c	*N. longiflora* Canvanilles		*Alatae* Goodspeed	6	67	30	40
TAB	a	*N. tabacum* L.		*Nicotiana* Don	13	74	30	130
GLAU	a	*N. glauca* Graham		*Noctiflorae* Goodspeed	22	139	13	155
NIC476	c	*N. noctiflora* Hooker		*Noctiflorae* Goodspeed	6	59	22	60
NIC507	c	*N. knightiana* Goodspeed		*Paniculatae* Goodspeed	6	121	13	90
NIC506	c	*N. solanifolia*		*Paniculatae* Goodspeed	9	141	21	150
NIC444	c	*N. acuminata* (Graham) Hooker var. *multiflora* (Phil) Reiche		*Petunioides* Don	5	43	14	28
NIC38	c	*N. quadrivalvis* Pursh var*. quadrivalvis*		*Polydicliae* Don	9	44	13	17
NIC521	c	*N. repanda* Willdenow ex Lehmann		*Repandae* Goodspeed	8	57	25	40
RUST	a	*N. rustica* L.		*Rustica* Don	4	53	19	95
Blab	b	*N. benthamiana*	Lab strain	*Suaveolentes* Goodspeed	6	43	16	20
BRA4	b	*N. benthamiana*	RA4 lab strain	*Suaveolentes* Goodspeed	6	43	16	20
B MN	a	*N. benthamiana*	RHUL lab strain	*Suaveolentes* Goodspeed	6	43	16	20
BNWA	b	*N. benthamiana*	North‐West Australia	*Suaveolentes* Goodspeed	6	43	11	14
B WA	b	*N. benthamiana*	West Australia	*Suaveolentes* Goodspeed	6	43	17	20
BQLD	b	*N. benthamiana*	Queensland	*Suaveolentes* Goodspeed	6	51	9	9
NIC6	c	*N. sylvestris* Spegazzini and Comes		*Sylvestres* Knapp	8	77	25	80
NIC479	c	*N. tomentosiformis* Goodspeed		*Tomentosae* Goodspeed	9	NA^d^	52	450
NIC406	c	*N. otophora* Grisebach		*Tomentosae* Goodspeed	10	NA^d^	51	450
NIC407	c	*N. tomentosa* Ruiz and Pavon var. *Leguiana* (J.F. Macbr.) Goodspeed		*Tomentosae* Goodspeed	12	NA^d^	52	450
NIC419	c	*N. glutinosa* L.		*Undulatae* Goodspeed	6	79	15	30

NA, leaves were harvested 6 months after germination, as flower emergence occurs after 9 months to 2 years.

^a^ Seeds (a) were available in‐house at Royal Holloway University or acquired from (b) www.herbalistics.com.au or (c) www.gbis.ipk‐gatersleben.de.

**Figure 1:**
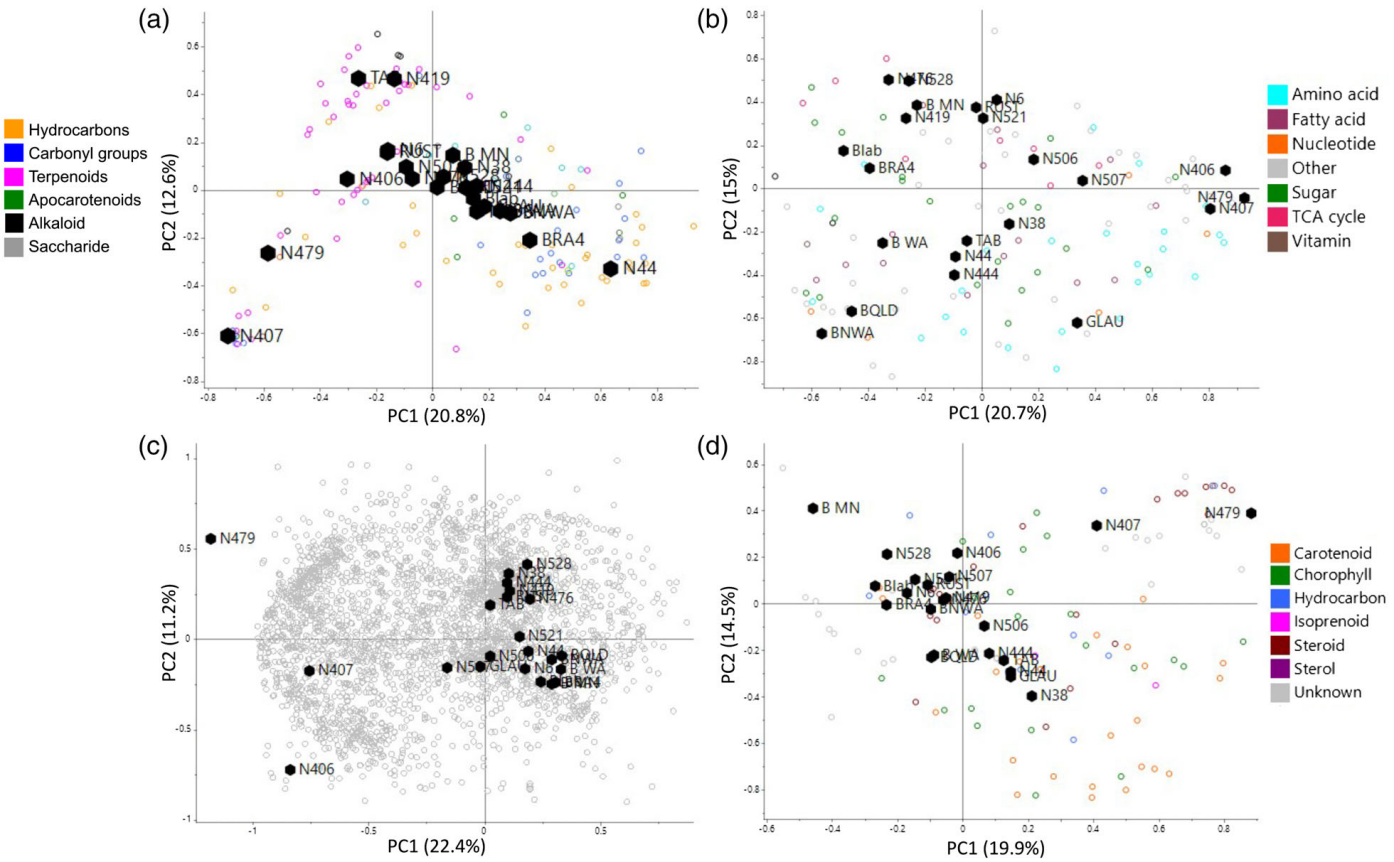
Metabolite analysis of leaves of a representative *Nicotiana* diversity panel. Data were separated into volatiles and semi‐volatiles analysed by SPME‐GC‐MS (a), primary metabolites analysed by GC‐MS (b), hydrophilic specialised metabolites analysed by metabolite profiling with LC‐MS (c) and hydrophobic specialised metabolites analysed by GC‐MS and UPLC‐PDA (d). Panels (a)–(c) include only identified metabolites coloured by chemical class (see individual legends). Unknown includes compounds present in the majority of samples which could not be identified. [Colour figure can be viewed at wileyonlinelibrary.com]

The core metabolome comprised primary metabolites such as central carbon metabolism, amino acids, nucleotides and fatty acids and common specialised metabolites such as sterols, photosynthesis‐related isoprenoids and hydrocarbons (Figure 1b,d). The other metabolites of the CCC, from here on referred to as specialised metabolites or metabolome, were mainly detected through analysis of volatiles by solid‐phase microextraction (SPME)‐GC‐MS and metabolite profiling of polar extracts by LC‐HRMS (Figure 1a,c). The specialised metabolites comprised a wide range of chemical classes, e.g. pyridine alkaloids, terpenoids, phenylpropanoids and flavonoids, and presented the largest proportion of chemical diversity, as previously reported (De Luca et al., 2012). All metabolites detected in the present study, except for unidentified molecular features, have been previously reported for individual *Nicotiana* species, mainly *N. tabacum* (Jassbi et al., 2017; Kaminski et al., 2020a). As previously reported for several *N. benthamiana* accessions, the present metabolite profiling did not detect any catechins or related/derived metabolites (Drapal et al., 2021b). This supports the hypothesis that the genus *Nicotiana* does not contain tannin synthesis, one of the major branches of the phenylpropanoid‐derived biosynthesis (Vogt, 2010).

Principal component analysis (PCA) of the *Nicotiana* metabolome highlighted that the three species of section *Tomentosae* Goodspeed were different from the other *Nicotiana* species, especially with regard to polar metabolites (Figure 1b,c). *Nicotiana otophora* (N406) was more closely located to the main cluster in the score plots for volatiles and hydrophobic specialised metabolites (Figure 1a,d). The similar trends between volatile data and organic extracts was to be expected as many volatiles are degradation productions from carotenoids, chlorophylls, steroids and fatty acids (Jassbi et al., 2017). Further statistical analysis was performed on the identified metabolite data set and the untargeted profiling and showed that 86 and 97% of the metabolites/features were significantly different between all the *Nicotiana* species. The chemical variation in the *Nicotiana* diversity panel showed low covariance between the metabolites leading to low eigenvalues. This resulted in approximately 35% of the variance in the data set being accounted for by the first two principal components for score plots of the core and specialised metabolomes (Figure 1). The low predictive ability (<10%) of the calculated PCA model corroborates the great metabolic diversity present in the genus *Nicotiana*, which varies in terms of both presence of metabolites and compositional differences (Worley & Powers, 2013).

PCA showed no separation based on ploidy (Figure 1). The alloploid species in the diversity panel include *N. benthamiana*, *N. rustica* L., *N. tabacum*, *N. repanda* Willdenow ex Lehmann and *N. quadrivalvis* Push var. *quadrivalvis*. The latter two species had parental genome donors from the *Trigonophyllae* section, which was not present in the diversity panel (Knapp et al., 2004). The first three species were compared to their diploid progenitors (Figure S2) and highlighted that *N. tabacum* and *N. rustica* had a closer metabolic similarity to their maternal ancestors *N. sylvestris* and *Nicotiana knightiana*, respectively. The same results were observed in a comparison of chloroplast genomes between parental genome donors and progeny and suggests a strong influence of the chloroplast‐derived regulatory factors on the leaf metabolome in *Nicotiana* species (Sierro et al., 2018; Yukawa et al., 2006). *Nicotiana benthamiana* showed a higher similarity to *Nicotiana acuminata* (Graham) Hooker var. *multiflora* (Phil) Reiche, a species of the *Petunioides* section. Previous genetic analysis speculated that *N. sylvestris* contained some introgressed DNA from the *Petunioides* section, before the hybridisation event with *Nicotiana noctiflora* (Schiavinato et al., 2020). Despite the similarities of progenies and progenitors, the alloploid species showed distinct metabolite profiles (Figure 1, Figure S2). This indicates that a range of changes associated with hybridisation occurred (e.g. altered transcriptional processes, stoichiometries of regulatory factors, interaction of duplicated genes and subgenome intermixing) and influenced the chemical composition of the alloploid progeny (Cheng et al., 2019; Daniell et al., 2016; Nieto Feliner et al., 2020; Schoenfelder & Fox, 2015).

### Database curation for the genus *Nicotiana*

**Figure 2:**
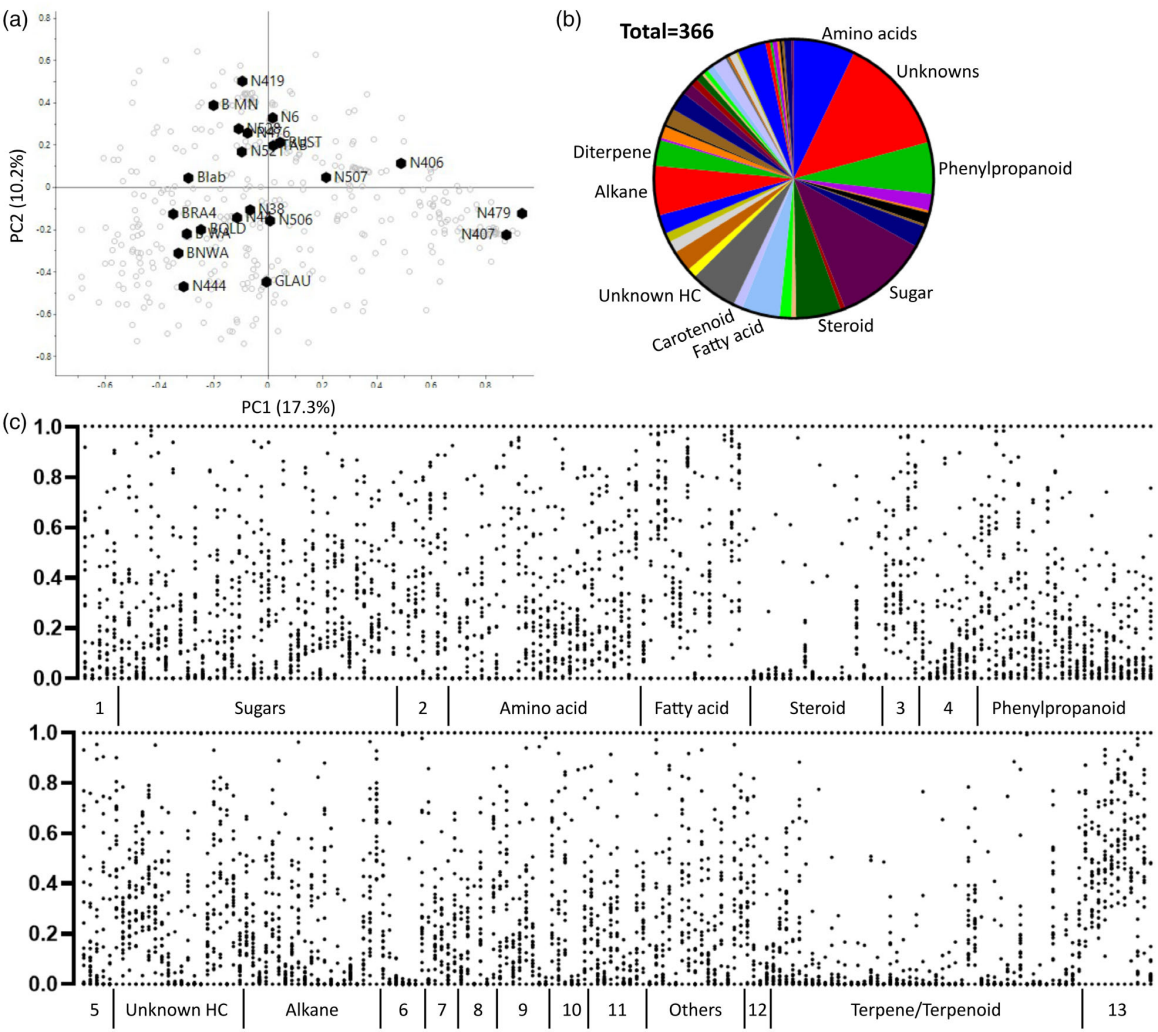
Quantitative display of annotated metabolites and recurring unknowns. (a) Biplot of *Nicotiana* diversity panel showing the average of six biological replicates. (b) Pie chart showing the distribution of chemical classes of the 366 annotated metabolites. Chemical classes with the largest number of metabolites are labelled. Full labels are available in Figure S2. (c) Average metabolite concentrations were rescaled between 0 (lowest conc.) to 1 (highest conc.) to display the distribution of metabolite levels within the diversity panel. Chemical classes: Unknown HC, unidentified hydrocarbons; Others include salicylic acid, pyrone, pyrrole, furans, resorcinols and sugars; 1, nucleotide; 2, TCA cycle; 3, sterol; 4, flavonoid; 5, alkaloid; 6, alkene; 7, acid; 8, alcohol; 9, aldehyde; 10, ketone; 11, ester; 12, apocarotenoid; 13, carotenoid/chlorophyll. [Colour figure can be viewed at wileyonlinelibrary.com]

Based on the present analysis, the database curated for *Nicotiana* species includes over 360 identified metabolites, with 32% comprising unidentified features from GC‐MS analysis. These unidentified features can be separated into unknowns level 3 (putatively characterised compound class) and unknowns level 4 (reoccurring spectral signal). The chemical compound classes of the metabolites are visualised as a pie chart (Figure 2b, Figure S3). The most prominent chemical classes were sugars, phenylpropanoids, amino acids, steroids, fatty acids, alkanes and unidentified/unknown hydrocarbons and included more than 19 and up to 40 individual metabolites. Jassbi et al. (2017) defined three main groups of specialised metabolites: terpenes, phenolics and nitrogen‐ or sulphur‐containing compounds, which comprised 87, 31 and seven identified metabolites, respectively. The number of identified terpenes was significantly higher than for phenolics due to availability of databases. The identification of phenolics from LC‐MS metabolite profiling is an intense manual process and relies on fragmentation patterns available in literature and authentic standards. Recent publications proposed methods for identification of phenolic acids and flavonoids of different plant species (Cerrato et al., 2020; Liu et al., 2019). However, these methods require specific analysis techniques and software. Furthermore, the phytochemical studies summarised by Jassbi et al. (2017) highlighted that the majority of phenylpropanoid‐derived compounds were detected in stem and roots.

All metabolites in the database were quantified relative to respective internal standards to report the dynamic range of each metabolite present in the genus *Nicotiana* (Table S1). These data were rescaled to facilitate visualisation of the metabolite data without the dynamic range (Figure 2c). The graph shows an obvious difference between core and specialised metabolome with an even distribution of the first (e.g. sugars, amino acids and fatty acids) and tight clusters of the latter (e.g. steroids and phenylpropanoids). The different distribution patterns indicate the importance of individual metabolites compared to metabolite classes for the metabolic regulation of *Nicotiana* species. An example are the volatile terpene/terpenoid‐derived chlorophylls and carotenoids. Chlorophylls and carotenoids are an essential feature of photosynthesis and have been detected in all species of the diversity panel. Derived volatiles, such as β‐ionone and caryophyllene, were only detected in a few *Nicotiana* species and represent a pathway (i) specific to individual *Nicotiana* species or (ii) partially related to a stress response and therefore not permanently present in all species.

**Figure 3:**
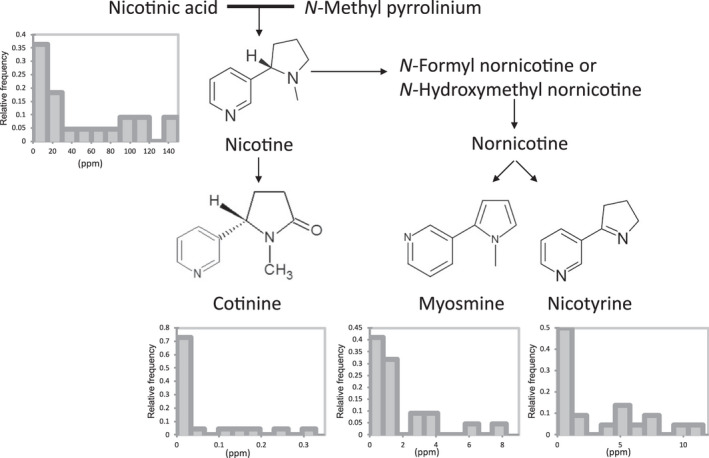
Content of alkaloids detected in the *Nicotiana* diversity panel. Histograms describing the frequency of alkaloid content (ppm relative to an internal standard) in the diversity panel are displayed next to the respective alkaloid name in the partial alkaloid pathway.

The metabolite data set including all of the identified metabolites was visualised as a score plot and showed very similar patterns to the individual score plots (Figure 2a). Additionally, the score plot was displayed as a dendrogram and compared to phylogenetic relationships inferred from multiple plastid DNA regions (Figure S4; Clarkson et al., 2004). The two phylogenetic trees showed different groupings of the *Nicotiana* species. This is a common occurrence between metabolite profiling, covering a wide range of metabolic pathways, and genotyping, which is typically based on a narrow selection of DNA regions and often not related to plant metabolism, e.g. the 16S rRNA gene (Drapal et al., 2020a, 2020b; Sirijan et al., 2020). However, the combination of genotyping and metabolomics can facilitate the elucidation of specific pathways, such as the evolution of nicotine biosynthesis in *N. tabacum* (Kajikawa et al., 2017; Xu et al., 2017) (Figure 3).

### Metabolites of interest

#### Nicotine and pyridine alkaloids

Nicotine, a pyridine alkaloid, is the most well‐known and well‐studied chemical present in *Nicotiana* species (Kajikawa et al., 2017). Pyridine alkaloid biosynthesis evolved in *Nicotiana* species as part of their biotic defence mechanism (Wink, 1988). Nicotine is the active compound in tobacco products and its use as a stimulant for humans dates back to around the first century BC (Mishra & Mishra, 2013). Adverse effects of nicotine and smoking on human health were scientifically reported as early as the 19th century (e.g. Anon, 1873). Other pyridine alkaloids produced by *Nicotiana* species, such as anatabine and anabasine, have recently been associated with beneficial medicinal properties for e.g. Alzheimer disease, chronic lymphocytic autoimmune (Hashimoto's) thyroiditis and obesity (Grebenstein et al., 2022; Schmeltz et al., 2014; Verma et al., 2015). The untargeted approach, used in the present study for volatile analysis, detected nicotine and four derived pyridine alkaloids: cotinine, nornicotine, myosmine and nicotyrine. A more targeted approach is needed to establish the full alkaloid composition, as previously published for 60 *Nicotiana* species (Kaminski et al., 2020a, 2020b; Saitoh et al., 1985). The diversity panel analysed showed a wide range of nicotine levels (0.3–140 ppm), and that the other pyridine alkaloid present in the majority of species was myosmine. This result was different to previous literature, which used a targeted analysis approach for pyridine alkaloids instead of volatile analysis by SPME‐GC‐MS from freeze‐dried leaves (Kaminski, et al., 2020a, 2020b; Saitoh et al., 1985). The highest levels of nicotine were detected in *N. rustica*, *N. tabacum* and *N. sylvestris* and the lowest levels (<1 ppm) in *Nicotiana glauca*, *Nicotiana longiflora*, *N. noctiflora* and *N. otophora*. The latter four species also had low levels (<1 ppm) of the other four alkaloids. The metabolite data highlighted a negative correlation of nicotine to precursors aspartic acid, cadaverine and glutamic acid as well as amino acids glutamine, GABA, threonine, serine, alanine and glycine and polyamine spermidine. Reduction of nicotine content has been previously associated with higher content of precursors (Ren et al., 2020). This implies that more assimilated nitrogen is available for amino acids and subsequently protein biosynthesis in *Nicotiana* plants with reduced pyridine alkaloid biosynthesis. These *Nicotiana* plants with reduced alkaloids are desirable as renewable biofactories from the perspective of (i) more N being available potentially for heterologous protein production and (ii) downstream processing which presently requires nicotine removal or grafting onto tomato (*Solanum lycopersicum*) (Fu et al., 2010).

#### Chlorogenic acids

**Figure 4:**
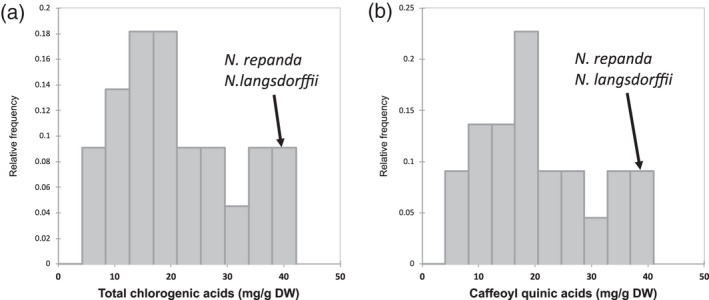
Content of total chlorogenic acids (a) and caffeoyl quinic acids (b) detected in the *Nicotiana* diversity panel. *Nicotiana* species with the highest content are labelled in the histograms.

Chlorogenic acids are phenolic compounds and comprise esters formed between quinic acid and a range of hydroxycinnamic acids. In plants, chlorogenic acids serve as precursors for lignin, which provides mechanical support and enables water transport, and function as defence molecules (Dixon & Barros, 2021; Hoffmann et al., 2004). The levels of chlorogenic acids differed by 10‐fold between the *Nicotiana* species analysed, e.g. *N. tomentosa* (4.3 mg g^−1^ dry weight [DW]) and *N. langsdorffii*/*N. repanda* (approximately 40 mg g^−1^ DW) (Figure 4a). The main type of chlorogenic acids in the diversity panel were caffeic acid esters (approximately 98%), followed by coumaric and ferulic acid esters in equal proportion (approximately 1%). All three isomers of caffeoyl quinates (neochlorogenic, chlorogenic and cryptochlorogenic acid) were detected in all *Nicotiana* species, as well as chlorogenic acid glycosides and dicaffeoyl quinates, and were summarised as caffeoyl quinic acids (Figure 4b). Esterification and glycosylation of hydroxycinnamic acid increases their water solubility and facilitates storage of the latter in the vacuole (Volpi E Silva et al., 2019). Furthermore, the two caffeic acids in dicaffeoyl quinate increase the number of hydroxy groups present, which is directly correlated to the increased antioxidant properties (Islam, 2006). Chlorogenic acid glycosides and dicaffeoyl quinates were present with an average of 1.5 and 0.8% of the caffeoyl quinate pool. Two *Nicotiana* species contained above average levels of these metabolites: *Nicotiana solanifolia* (approximately 18% chlorogenic acid glycosides) and *N. knightiana* Goodspeed (approximately 5% dicaffeoyl quinates).

Chlorogenic acids are important functional compounds in the human diet and one of the most abundant polyphenols in fruits, vegetables and coffee. Chlorogenic acids provide multiple biological activities, including antioxidant and anti‐carcinogenic activities, reduction of coronary heart disease and inflammation, cytotoxicity and anti‐human immunodeficiency virus‐1 activity (Chen et al. 2012; dos Santos et al., 2006). However, chlorogenic acids are sensitive to heat and light and easily oxidised. These physical properties can be improved through glycosylation and are more amenable for industrial application and food supplements (Nam et al., 2017). The presence of glycosylated chlorogenic acids in *Nicotiana* indicates the presence of the respective pathways, which could be modified for enhanced biosynthesis.

#### Squalene and sterols

**Figure 5:**
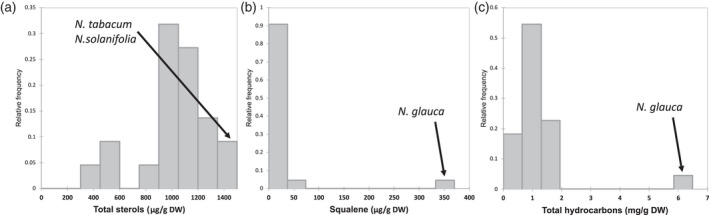
Content of total sterols (a), squalene (b) and total hydrocarbons (c) detected in the *Nicotiana* diversity panel. *Nicotiana* species with the highest content are labelled in the histograms.

Sterols are an important part of the plant and human metabolism as structural components of membranes, for developmental signalling in plants and as precursors for steroidal hormones in humans. Phytosterols are also known to reduced blood cholesterol levels besides many more beneficial properties for human health (Häggström & Richfield, 2014; Lindemann, 2015; Ostlund, 2002). Squalene is the product of the first committed intermediate in sterol biosynthesis (Nguyen et al., 2013; Suza & Chappell, 2016). The levels of squalene in the majority of *Nicotiana* species were approximately 11.5 μg g^−1^ DW, except for *N. glauca* Graham, which contained 370 μg squalene per gram DW and a total sterol content of 1.3 mg g^−1^ DW (Figure 5b). The squalene levels showed no correlation to total sterol levels within the *Nicotiana* diversity panel. The total sterol content in the diversity panel ranged from approximately 0.4 to 1.4 mg g^−1^ DW and *N. tabacum* and *N. solanifolia* had the highest levels (Figure 5a). The composition of sterols consisted of β‐sitosterol, stigmasterol, campesterol and cholesterol at varying percentage (1–72%) and the latter two were not present in all *Nicotiana* species. Sterols are hydrophobic metabolites and can be easily separated from the toxic, hydrophilic alkaloids (e.g. nicotine), which is of great importance for multiple uses of the phytosterol extracts such as food supplements and precursor for steroidal bioconversion (Batth et al., 2020; Tarkowská, 2019).

#### Hydrocarbons

Renewable sources for biofuel production are a prominent topic worldwide. The most established routes are bioethanol production from corn (*Zea mays*) and sugar cane (*Saccharum spp*) and biodiesel production from vegetable oils. Recently, the focus shifted to non‐food crops, which can grow on marginal lands, to allocate the nutrient‐rich agricultural lands for cultivation of food crops (Hood, 2016). *Nicotiana tabacum* has similar levels of non‐structural sugars and lower levels of lignin compared to energy crops (Barla & Kumar, 2019). A significant amount of plant waste is produced during the processing of leaves for tobacco products and includes stalks and leaves with an above average biomass compared to other *Nicotiana* species (Table 1). These waste products could be utilised for biomass conversion to biofuels. Despite the promising use of tobacco waste, the cultivation of *N. tabacum* solely for energy production would not be beneficial as it requires nutrient‐rich lands. On the contrary, *N. glauca* can be cultivated on marginal lands under extreme environmental conditions and produces a significantly higher amount of long‐chain hydrocarbons (approximately 6 mg g^−1^ DW) compared to other *Nicotiana* species (Figure 5c). Leaf material of *N. glauca* contained mainly hentriacontane (approximately 72%) and hexacosanol (approximately 17%), whereas other *Nicotiana* species contained an equal amount of about seven hydrocarbons with a carbon chain of 29 and higher. For *N. glauca*, the hydrocarbons can be extracted with a rapid solvent procedure, which separates the energy‐dense products from the biomass containing additional structural and non‐structural hydrocarbons including terpenoids (Mortimer et al., 2012).

#### Precursor pathways for engineering

**Figure 6:**
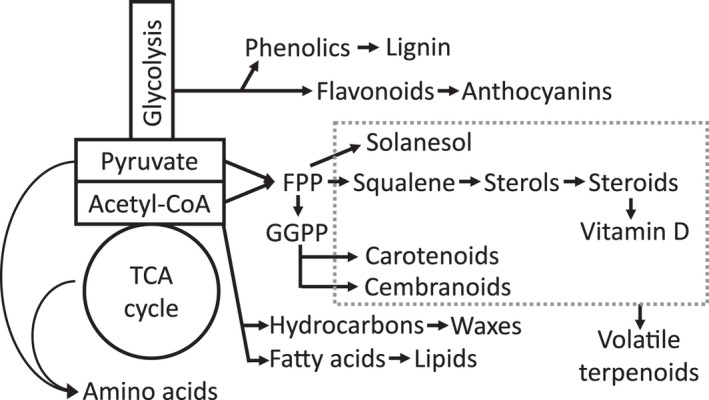
Biosynthetic pathways for high‐value end‐products. Grey box indicates metabolite groups classed as terpenoids.

*Nicotiana* species contain a large number of biologically active natural products used in agriculture, foods, supplement, cosmetics, pharmaceuticals and petrochemistry (Jassbi et al., 2017; Rodgman & Perfetti, 2013). The present study presents an overview of the type and amount of precursors and end‐products present in the genus *Nicotiana* (Figure 6). Based on the chemical composition, specific species can be used to study pathways and facilitate production of non‐endogenous high‐value compounds (Table 2). One of the most prevalent precursors for high‐value compounds is isopentenyl pyrophosphate (IPP), derived from the cytosolic mevalonate pathway and the plastidial non‐mevalonate pathway. Next to the already described sterols and steroids, IPP‐derived farnesyl pyrophosphate (FPP) is also converted to solanesol and cembranoids, known for their nutritional and medicinal properties (Rowland et al., 1956; Sui et al., 2018; Zhang et al., 2021). These four end‐products are part of the chemical class terpenes along with a large number of other metabolites included in foodstuff and important for medicinal purposes and petrochemistry (Delatte et al., 2018; Lee et al., 2019; Reed & Osbourn, 2018; Wu et al., 2012). Other compounds derived from IPP and FPP have been transiently expressed in *N. benthamiana*, e.g. the malaria drug artemisinin and the cancer drug taxane (Li et al., 2019; Wang et al., 2016; Xu et al., 2016). Specialised analytical platforms are required for the detection and quantification of IPP and FPP and were not included in the present metabolite profiling. However, the increased quantity of detected end‐products in some *Nicotiana* species (e.g. *N. glauca*, *N. quadrivalvis*, *N. tabacum* and *N. solanifolia*) suggests an increased metabolic flow through the mevalonate and non‐mevalonate pathways, which can be diverted to new engineer pathways.

**Table 2:** Endogenous precursors and high‐value products present or previously expressed in *Nicotiana* species. Species from the present diversity panel with the highest amount of precursor are listed including the amount present in freeze‐dried material

Precursor	Product	Application	References	Species	Content (μg g^−1^ DW)
Squalene	Steroids	Medicinal	Spivak et al. (2010)	*N. glauca*	369
Sterols	Vitamin D	Vitamin deficiency	Skliar et al. (2000)	*N. tabacum* *N. solanifolia*	1400
Phytoene	Carotenoids	Nutrition, vitamin deficiency	Majer et al. (2017), Llorente et al. (2020)	*N. rustica*	196
β‐Carotene	Ketocarotenoids	Nutrition, agriculture	Nogueira et al. (2017)	*N. acuminata multiflora*	1161
	Saffron apocarotenoids	Medicinal, foodstuff	Martí et al. (2020), Demurtas et al. (2018)		
Phenylalanine	Shikimic acid	Medicinal	Nagasawa (1958), Scalabrin et al. (2016)	*N. glauca*	655
Shikimic acid	Anthocyanins	Nutrition, agriculture	Fresquet‐Corrales et al. (2017), Lloyd et al. (1992), Appelhagen et al. (2018)	*N. repanda*	966
Acetyl‐CoA/ fatty acids	Lipids	Biofuels	Pouvreau et al. (2020), Reynolds et al. (2015), Tian et al. (2020)	*N. sylvestris*	6320

Abbreviations: IPP, isopentenyl pyrophosphate; FPP, farnesyl pyrophosphate; GGPP, geranylgeranyl pyrophosphate; N/M, not measured in the present study.

## CONCLUSION

In order to reach global targets on net zero emissions, manufacturing practices will in the future need to be sustainable. Therefore, it is important that humanity knows the chemicals available in present plant‐based cell factories, which can be utilised to replace fossil‐fuel‐derived or chemical synthesis in the future. The genus *Nicotiana* is a rich source of chemical diversity. The present study aimed to capture this chemical diversity using multi‐platform analytical approaches. The data set created represents the CCC of *Nicotiana*, available to date. The data set created contains over 360 annotated chemical features common to all *Nicotiana* species or unique to individual sections or species. The data produced also contained hundreds of chemical features which could not be identified. The annotation of these compounds relies on the current and future development of metabolite databases and production of authentic standards (Cerrato et al., 2020; Kind & Fiehn, 2007; Liu et al., 2019). One of these databases is NicotianaCyc, which contains experimentally verified and manually curated metabolic data for the *Nicotiana* genus (Foerster et al., 2021).

A comparison of the dendrogram based on the annotated metabolite data and genotyping highlighted that the different levels of cellular regulation are not always representative of each other. For example, the data inferred that all *Nicotiana* species analysed contain the genetic information for sterol biosynthesis, including squalene synthase. Only *N. glauca* produced above average levels of squalene, indicating a different regulatory mechanism beyond transcription. The characterisation of the *Nicotiana* genus provides crucial information to elucidate the evolution of metabolite pathways and establishes a baseline metabolome, which can be improved through physiological and elicitation methods (Kaminski et al., 2020a). Many compounds require a multi‐step synthesis by multiple enzymes and are too complex to be produced *in vitro* or in bacterial/yeast cell factories. *Nicotiana* plants already contain a large chemical diversity, have a high biomass and are amenable to New Plant Breeding Techniques. Hence, *Nicotiana* species, especially *N. tabacum*, are an ideal chassis to produce multiple high‐value chemicals in a renewable manner using biorefining cascades.

## EXPERIMENTAL PROCEDURES

The diversity panel of *Nicotiana* species was grown twice with three biological replicates each. The first set of replicates were sown at the end of August and the second set of replicates were sown in the consecutive mid‐December. Seeds were germinated on Levington® Advance Pot&Bedding Compost F2 + Sand (ICL Specialty Fertilisers, Ipswich, UK). Once plantlets consisted of three to four leaves, they were transferred to individual pots (5 L) with Levington® Advance Pot&Bedding Compost M3 (ICL Specialty Fertilisers). Plants were grown in a glasshouse at 24°C under supplementary lighting (16 h light/8 h dark cycle) and supplemented with Universol® Blue (ICL Specialty Fertilisers) once a week as described previously (Drapal et al., 2021a).

At the emergence of flower buds, two fully expanded leaves were harvested per biological replicate and immediately frozen in liquid nitrogen. The samples were stored at −80°C until all species of one set were sampled and then lyophilised. Dried leaves were ground to a fine powder with a TissueRuptor (Qiagen, Manchester, UK) and a portion of the ground powder (10–11 mg) was weighed out.

The extracts for three different analytical platforms were extracted with one methanol/water/chloroform separation method as previously described (Drapal et al., 2020b). The polar phase was aliquoted for metabolite profiling (100 μl) by UPLC‐ESI‐QToF 6560 (Agilent Technologies, Stockport, UK) and for metabolite profiling (150 μl) by GC‐MS detection (GC‐MSD) in splitless mode (Agilent Technologies) as previously described (Drapal et al., 2021b; Price et al., 2016). The extracts were spiked with internal standards genistein (1 μg) and d4‐succinic acid (10 μg). An aliquot (700 μl) of the non‐polar phase was dried down, resuspended in ethylacetate/acetonitrile (200 μl) and analysed (3 μl injection) by UPLC‐PDA (Waters™, Wilmslow, UK) as previously described (Drapal et al., 2020b). The resuspended samples were dried down again immediately after injection, spiked with d27‐myristic acid (10 μg) and analysed by GC‐MSD as described for the polar extract. All samples for GC‐MSD were derivatised prior to analysis as previously described (Price et al., 2016).

For volatile analysis, freeze‐dried and ground leaf samples (50 mg) were weighed into a headspace vial and spiked with d8‐acetophenone (21.1 μg). Analysis was performed with a Gerstel Multi Purpose Sampler (Gerstel) coupled with a GC‐MSD 5977B (Agilent Technologies) and a previously published temperature gradient of 50–260°C (Ma et al., 2012). The incubation was performed at 100°C for 5 min, followed by 30 min sample extraction with a DVB/CAR/PDMS fibre (50/30 μm) and desorption at 250°C for 6 min.

Data processing was performed with AMDIS (v2.71, NIST) and an in‐house library for GC‐MS files and with Agilent Profinder (v10.0 SP1; Agilent Technologies, Inc.) for LC‐MS files. The molecular features of LC‐MS files were then compared to an in‐house library based on retention time and mass spectrum. Database NIST11 (http://chemdata.nist.gov/mass‐spc/ms‐search/) was used for identification of GC‐MS compounds not present in the in‐house library comprised of authentic standards and published literature (Ncube et al., 2014). Identification of metabolites was composited into an in‐house library specific for *Nicotiana* species and used to annotate the metabolites in the data table. The metabolites were then quantified relative to the internal standard and the DW of the sample. For SPME‐GC‐MS analysis, the volatiles were only quantified relative to the internal standard.

PCA and hierarchical clustering analysis were performed with Simca® (17.0.0.24543, Sartorius Stedim Data Analytics AB) and significant differences were calculated with Metaboanalyst including Pareto scaling and analysis of variance (Xia & Wishart, 2016). All other graphs were created with GraphPad Prism (v.9.1.0, GraphPad Software, LLC).

## CONFLICT OF INTEREST

The authors declare no conflict of interest.

## AUTHOR CONTRIBUTIONS

MD, EMAE and PDF designed the study; MD performed the experiments and data analysis; MD, EMAE and PDF wrote the manuscript.

## Supporting information


**Table S1** Database of metabolite concentration range present in *Nicotiana*.

**Table S2** Metabolite profiling of a *Nicotiana* diversity panel.

**Figure S1** PCA score plot indicating seasonal effects on metabolism. Analysis is based on metabolite profiling data generated by LC‐MS. Varieties are colour‐coded (see legend) and seasons are indicated as dots (summer) and boxes (winter).

**Figure S2** Comparison of metabolite data of alloploid species *N. rustica* (a), *N. tabacum* (b) and *N. benthamiana* (c) to descendants of their diploid progenitors. Data from Table S2 are represented as average values of six biological replicates in a heatmap with hierarchical clustering of *Nicotiana* species and metabolites.

**Figure S3** Pie chart showing the distribution of chemical classes of the 374 annotated metabolites. More detailed visualisation of Figure 2b with a complete legend, including the number of compounds comprised in each chemical class.

**Figure S4** Dendrograms based on (a) the metabolite data generated in the present study and (b) multiple plastid DNA regions (Clarkson et al., 2004).

## Data Availability

All relevant data can be found within the manuscript and its supporting materials. Unprocessed data can be accessed at DOI: 10.17632/rhnxrfzm6n.2.
