# Supplementary material for: The chemotype core collection of genus Nicotiana
Source: Plant J. 2022 Apr 7;110(5):1516–28. doi: 10.1111/tpj.15745 (PMC9321557; doi:10.1111/tpj.15745)
Supplement: Supplementary file 3 — Figure S1 PCA score plot indicating seasonal effects on metabolism. Analysis is based on metabolite profiling data generated by LC‐MS. Varieties are colour‐coded (see legend) and seasons are indicated as dots (summer) and boxes (winter). [file TPJ-110-1516-s002.pdf]

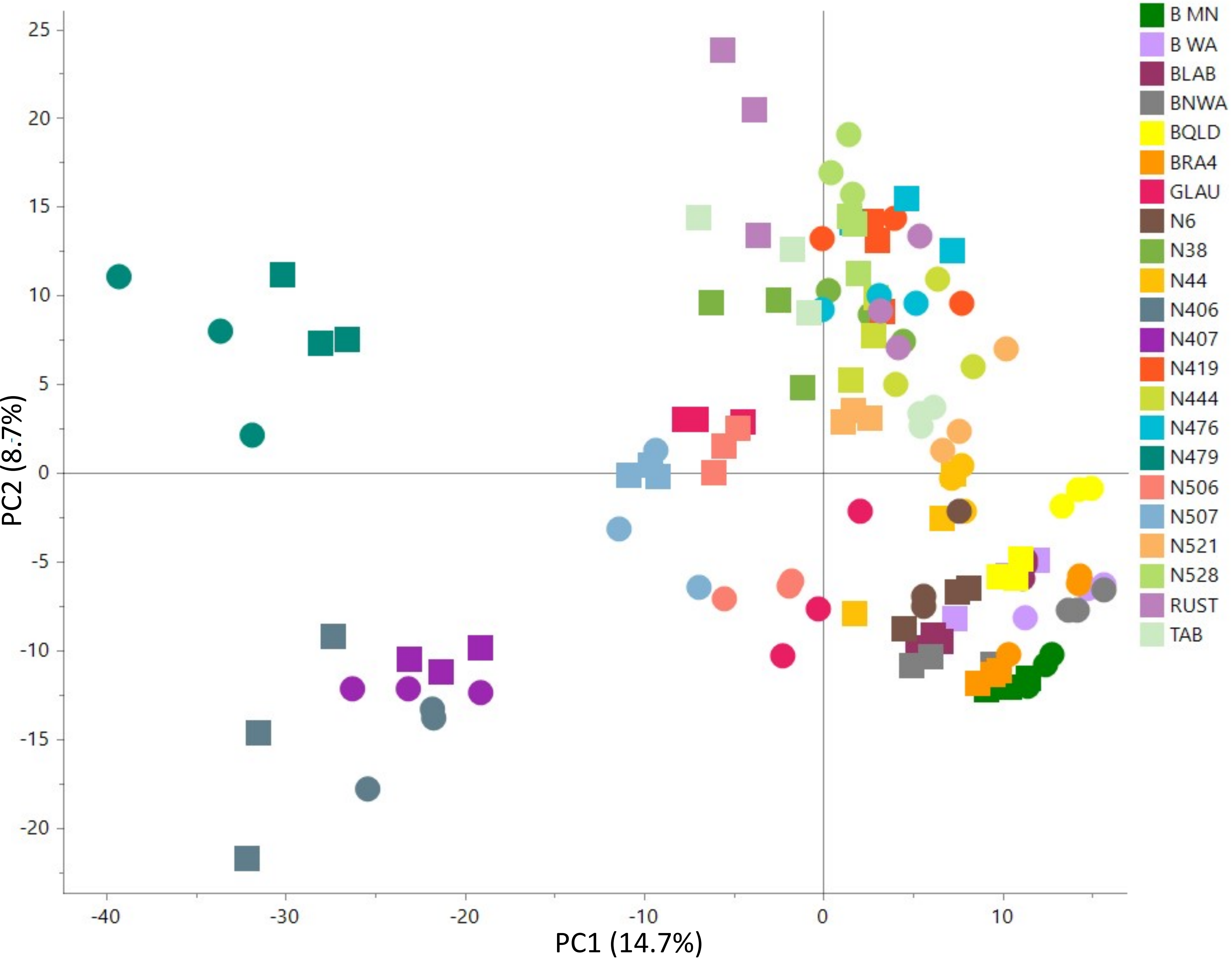

Figure S1. PCA score plot indicating seasonal effects on metabolism. Analysis is based on metabolite profiling data generated by LC-MS. Varieties are colour coded, see legend and seasons are indicated as dots (summer) and boxes (winter).
