# Supplementary material for: The chemotype core collection of genus Nicotiana
Source: Plant J. 2022 Apr 7;110(5):1516–28. doi: 10.1111/tpj.15745 (PMC9321557; doi:10.1111/tpj.15745)

Figure 2. Comparison of metabolite data of alloploid species *N. rustica* (A), *N. tabacum* (B) and *N. benthamiana* (C) to descendants of their diploid progenitors. Data from Table S2 was represented as average values of six biological replicates in a heatmap with hierarchical clustering of *Nicotiana* species and metabolites.

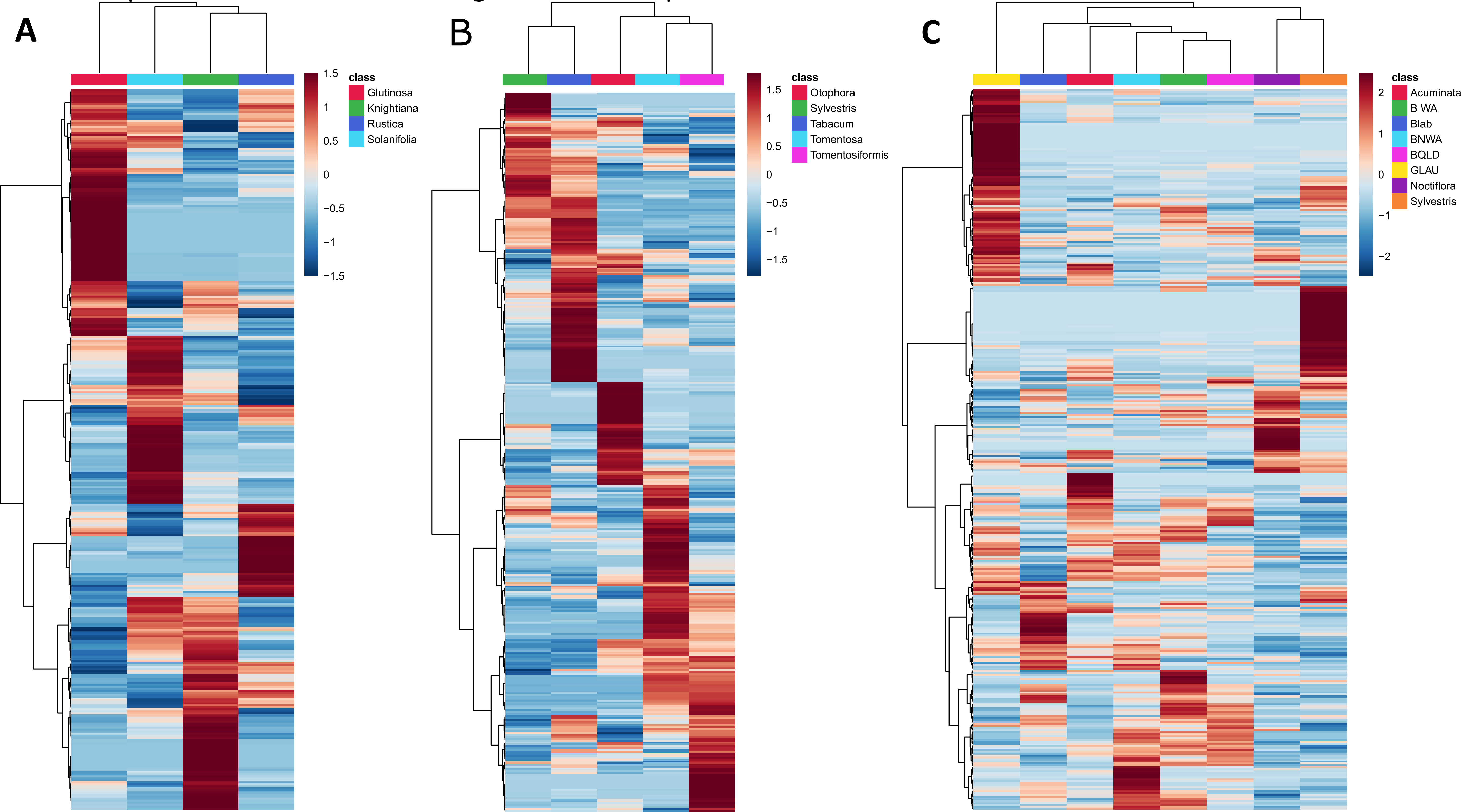

Supplement: Supplementary file 4 — Figure S2 Comparison of metabolite data of alloploid species N. rustica (a), N. tabacum (b) and N. benthamiana (c) to descendants of their diploid progenitors. Data from Table S2 are represented as average values of six biological replicates in a heatmap with hierarchical clustering of Nicotiana species and metabolites. [file TPJ-110-1516-s001.pdf]
