# Supplementary material for: The chemotype core collection of genus Nicotiana
Source: Plant J. 2022 Apr 7;110(5):1516–28. doi: 10.1111/tpj.15745 (PMC9321557; doi:10.1111/tpj.15745)
Supplement: Supplementary file 5 — Figure S3 Pie chart showing the distribution of chemical classes of the 374 annotated metabolites. More detailed visualisation of Figure 2b with a complete legend, including the number of compounds comprised in each chemical class. [file TPJ-110-1516-s004.pdf]

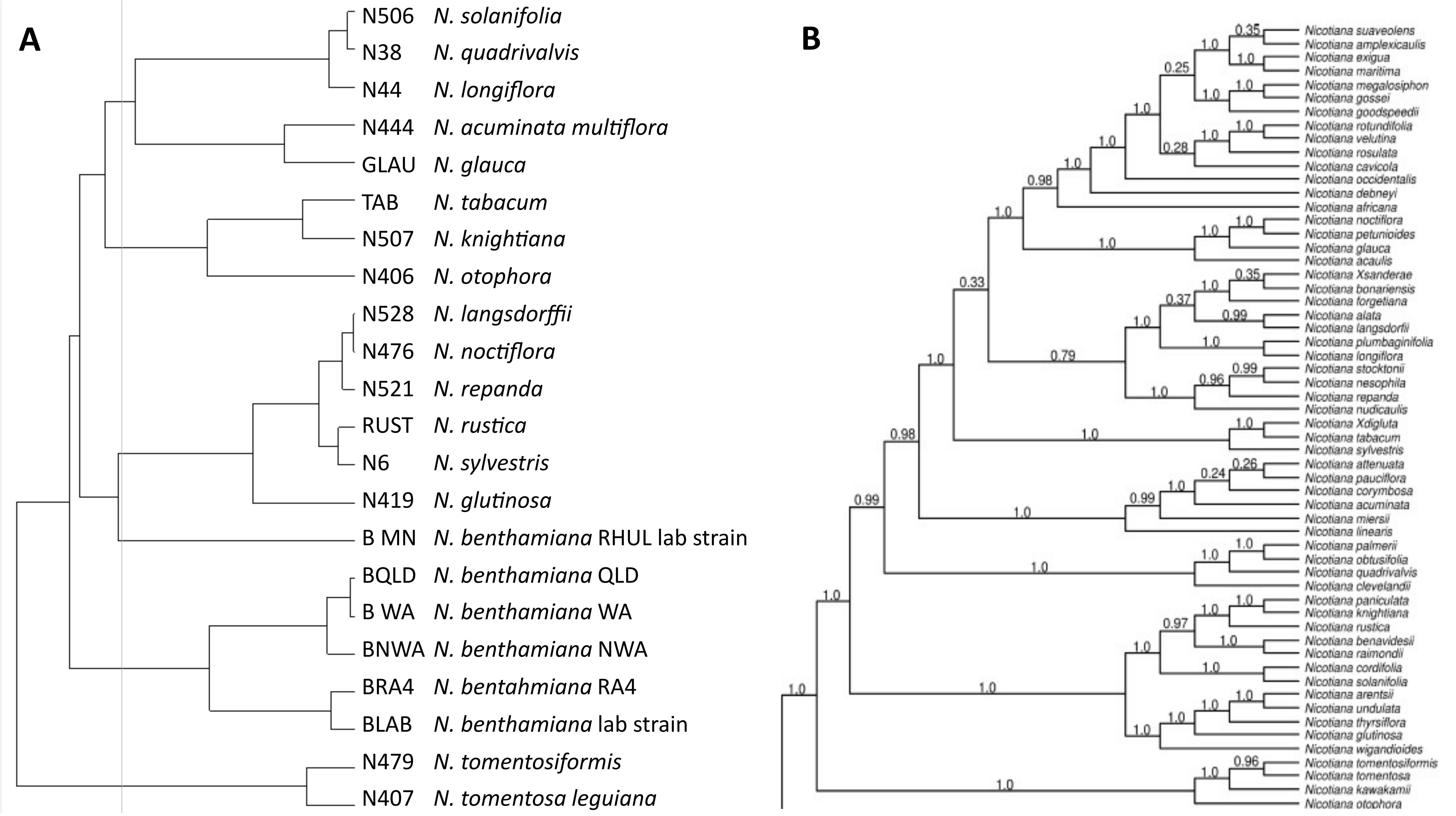


Figure S3. Dendrograms based on (A) the metabolite data generated in the present study and (B) multiple plastid DNA regions (figure altered from Fig. 2 (Clarkson *et al.* 2004)).
