# Supplementary material for: The chemotype core collection of genus Nicotiana
Source: Plant J. 2022 Apr 7;110(5):1516–28. doi: 10.1111/tpj.15745 (PMC9321557; doi:10.1111/tpj.15745)
Supplement: Supplementary file 6 — Figure S4 Dendrograms based on (a) the metabolite data generated in the present study and (b) multiple plastid DNA regions (Clarkson et al., 2004). [file TPJ-110-1516-s006.pdf]

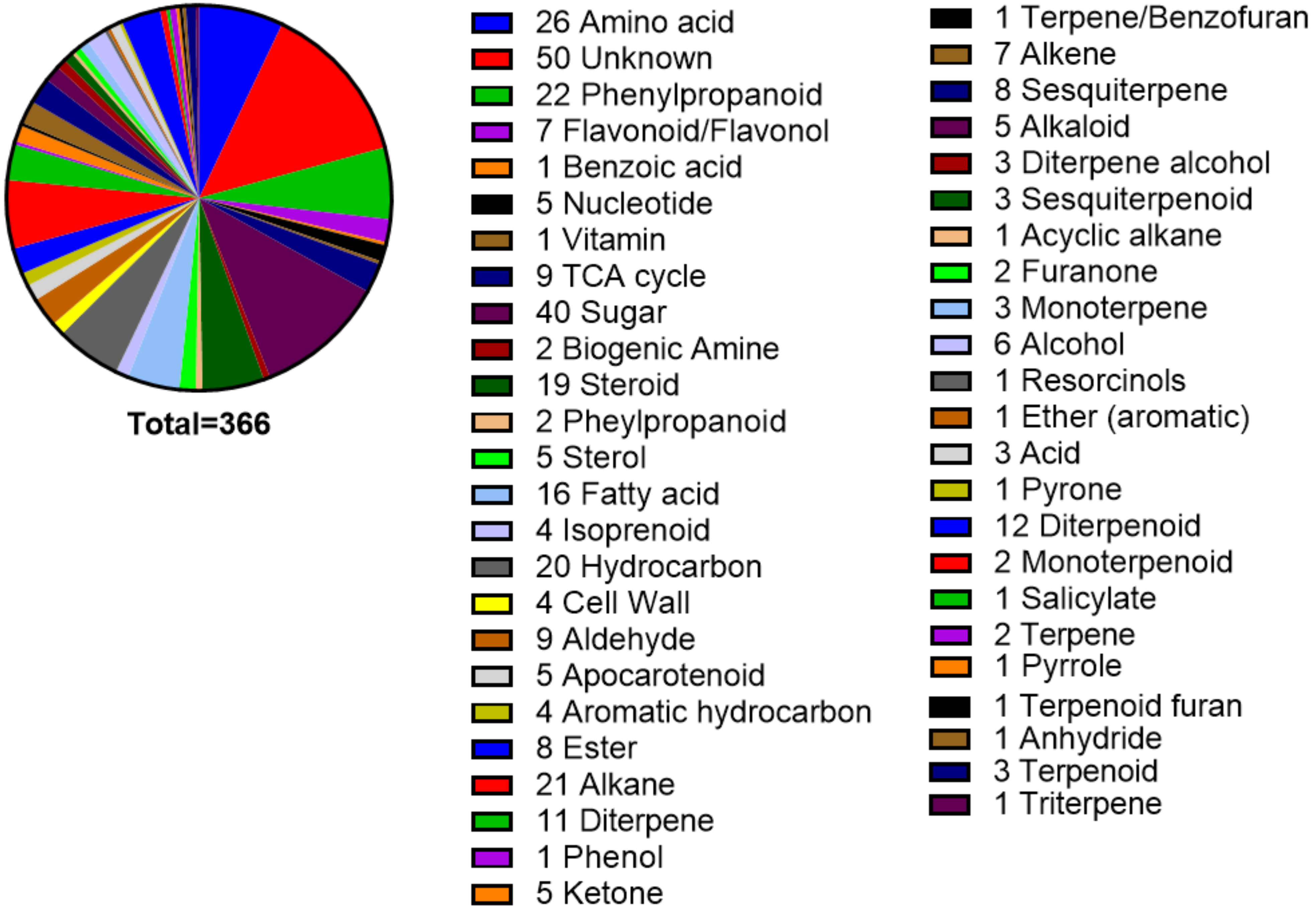

Figure S2. Pie chart showing the distribution of chemical classes of the 374 annotated metabolites. More detailed visualization of Figure 2B with a complete legend, including the number of compounds comprised in each chemical class.
